# Rectal cancer masquerading as submucosal tumors: endoscopic submucosal dissection uncovers the reality

**DOI:** 10.1055/a-2512-3840

**Published:** 2025-02-26

**Authors:** Zhixia Dong, Xiangyun Zhao, Bo Tian, Yueqin Qian, Xinjian Wan

**Affiliations:** 1 Digestive Endoscopic Center, Shanghai 6th Peoples Hospital Affiliated to Shanghai Jiaotong University School of Medicine, Shanghai, China

Rectal cancer typically exhibits distinctive endoscopic and radiological features 1 2 . We report an unusual and rare case of rectal cancer that mimicked submucosal tumors, which was accurately diagnosed using endoscopic submucosal dissection (ESD).

A 58-year-old woman presented to our hospital with complaints of fresh rectal bleeding. Colonoscopy examination revealed two submucosal tumor-like elevated lesions with smooth surface in the lower rectum, approximately 0.5–1.0 cm in size ( Fig. 1 **a, b** ). Endoscopic ultrasonography showed hypoechoic lesions in the submucosa, without invasion of the muscularis propria ( Fig. 1 **c, d** , Video 1 ). Magnetic resonance imaging demonstrated a nodule on the left side of the pelvic cavity, adjacent to the sigmoid colon, with a size of 1.5 cm ( Fig. 2 ), which was suspected of being benign. Consequently, the two lesions were initially suspected of being neuroendocrine tumors.

**Fig. 1:**
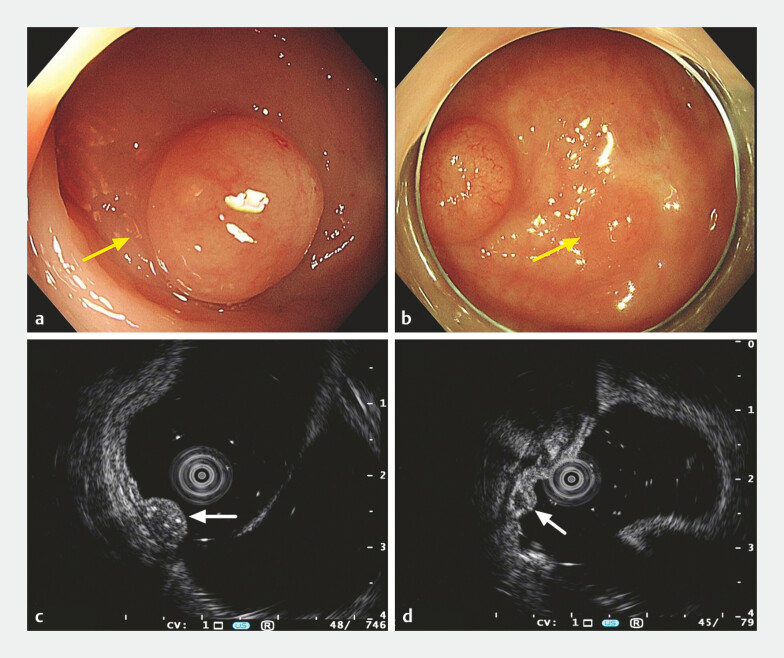
Examination and imaging of the lesion. **a, b** Colonoscopy showed two submucosal tumor-like elevated lesions with smooth surface in the lower rectum, approximately 0.5–1.0 cm in size (yellow arrows). **c, d** Endoscopic ultrasonography showed hypoechoic lesions located in the submucosa, without invasion of the muscularis propria (white arrows).

Rectal cancer masquerading as submucosal tumors and revealed by endoscopic submucosal dissection.Video 1

**Fig. 2:**
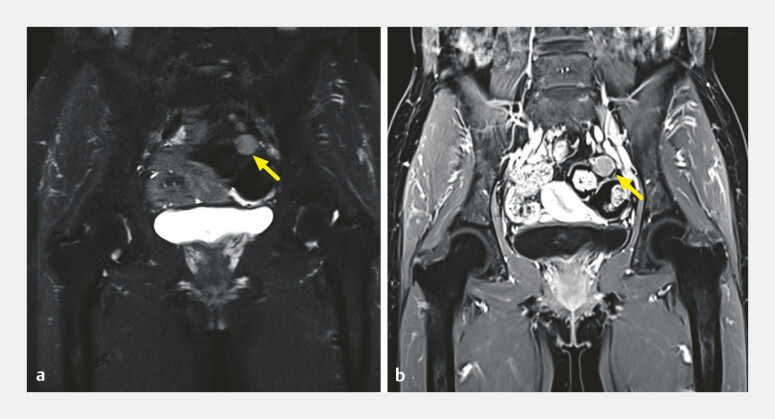
Abdominal magnetic resonance imaging (MRI). **a** A nodule with low signal intensity was visible on the left side of the pelvic cavity on T1-weighted MRI (yellow arrow). **b** Contrast-enhanced images on T2-weighted fat-suppressed MRI showed uniform enhancement (yellow arrow).

After obtaining informed consent, ESD was performed for complete en bloc resection to confirm the diagnosis ( Fig. 3 **a** , Video 1 ). Unexpectedly, histological examination of the resected specimen revealed adenocarcinoma of the rectum, with an invasion depth of approximately 3 mm. Histological results indicated that both of the tumors were situated in the submucosa, accompanied by focal involvement of the mucosal lamina propria. Notably, no tumorigenic changes in the surface glands were observed ( Fig. 3 **b, c** ), which also led to the tumors presenting as submucosal tumors. Immunohistochemistry further confirmed the diagnosis of primary rectal adenocarcinoma ( Fig. 4 ).

**Fig. 3:**
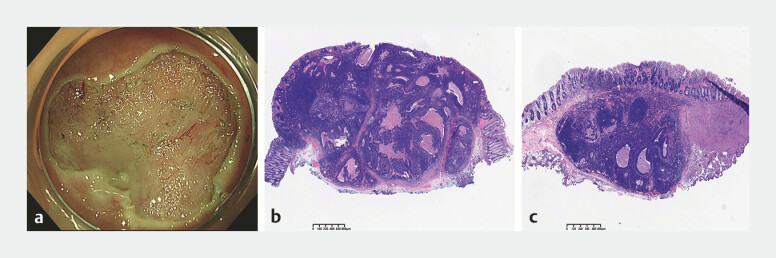
The resected specimen. **a** The mucosal defect immediately after endoscopic submucosal dissection. **b, c** Histological images of the resected specimen stained with hematoxylin and eosin showed adenocarcinoma situated in the submucosa, with an invasion depth of approximately 3 mm, while no tumorigenic changes were observed in the surface glands.

**Fig. 4:**
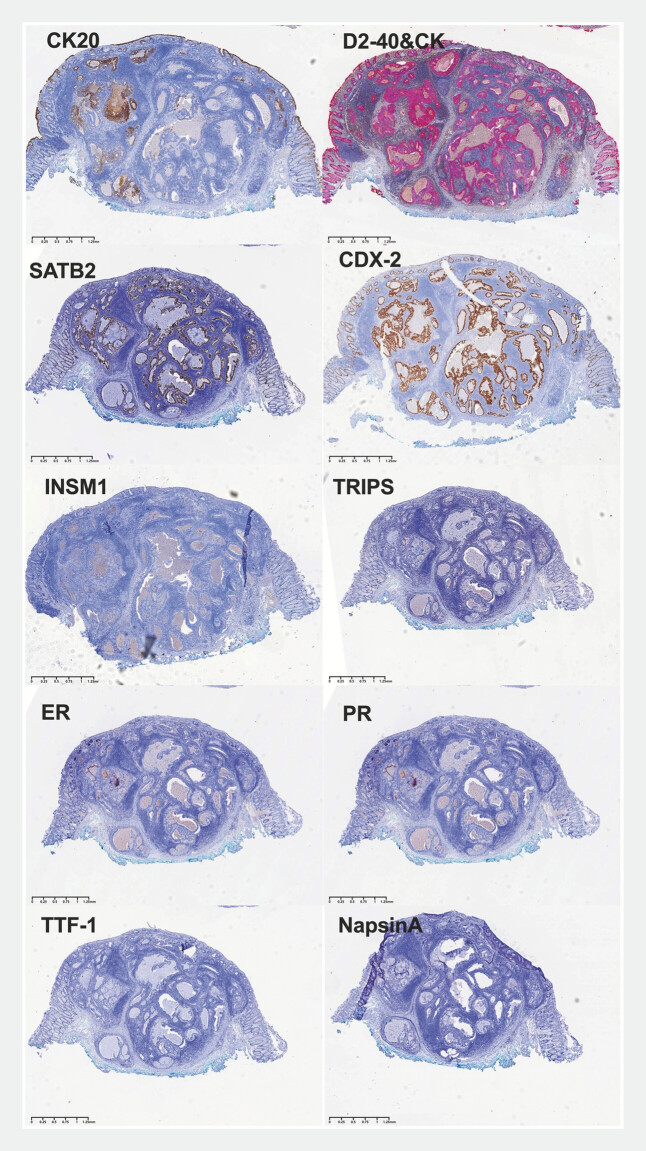
Immunohistochemical analysis confirmed the diagnosis of primary rectal adenocarcinoma, with positive specific markers for colorectal adenocarcinoma, including CK20, CDX-2, and SATB2. However, the immunohistochemical markers for lung adenocarcinoma (TTF-1 and NapsinA), breast adenocarcinoma (ER, PR, and Trips), and neuroendocrine tumors (INSM1) were all negative. There was partial positivity for CK, and D2–40 was negative.

This finding was surprising given the benign appearance of the lesion on imaging studies and the initial clinical suspicion of neuroendocrine tumors. This case underscores the importance of histological confirmation in the diagnosis of rectal lesions, even when imaging suggests benignity. It serves as a reminder that rectal cancer can sometimes exhibit atypical features, mimicking submucosal tumors or other benign conditions. Thus, maintaining a high index of suspicion and conducting thorough histological examination are crucial to avoid misdiagnosis and ensure appropriate treatment.

Endoscopy_UCTN_Code_CCL_1AD_2AB

## Notes

Endoscopy E-Videos https://eref.thieme.de/e-videos
*E-Videos* is an open access online section of the journal *Endoscopy* , reporting on interesting cases and new techniques in gastroenterological endoscopy. All papers include a high-quality video and are published with a Creative Commons CC-BY license. Endoscopy E-Videos qualify for HINARI discounts and waivers and eligibility is automatically checked during the submission process. We grant 100% waivers to articles whose corresponding authors are based in Group A countries and 50% waivers to those who are based in Group B countries as classified by Research4Life (see: https://www.research4life.org/access/eligibility/ ). This section has its own submission website at https://mc.manuscriptcentral.com/e-videos .
